# Glucose transporter‐1 deficiency syndrome with extreme phenotypic variability in a five‐generation family carrying a novel *SLC2A1* variant

**DOI:** 10.1111/ene.16325

**Published:** 2024-05-27

**Authors:** Alessia Giugno, Elena Falcone, Francesco Fortunato, Ilaria Sammarra, Radha Procopio, Monica Gagliardi, Alessia Bauleo, Laura de Stefano, Iolanda Martino, Antonio Gambardella

**Affiliations:** ^1^ Department of Medical and Surgical Sciences, Institute of Neurology University Magna Græcia Catanzaro Italy; ^2^ BIOGENET–Medical and Forensic Genetics Laboratory Cosenza Italy; ^3^ Department of Medical and Surgical Sciences, Neuroscience Research Center Magna Graecia University Catanzaro Italy

**Keywords:** childhood absence epilepsy, GLUT1 deficiency syndrome, hippocampal sclerosis, missense variant, *SLC2A1* gene

## Abstract

**Background and purpose:**

Glucose transporter‐1 (GLUT1) deficiency syndrome (GLUT1‐DS) is a metabolic disorder due to reduced expression of GLUT1, a glucose transporter of the central nervous system. GLUT1‐DS is caused by heterozygous *SLC2A1* variants that mostly arise de novo. Here, we report a large family with heterogeneous phenotypes related to a novel *SLC2A1* variant.

**Methods:**

We present clinical and genetic features of a five‐generation family with GLUT1‐DS.

**Results:**

The 14 (nine living) affected members had heterogeneous phenotypes, including seizures (11/14), behavioral disturbances (5/14), mild intellectual disability (3/14), and/or gait disabilities (2/14). Brain magnetic resonance imaging revealed hippocampal sclerosis in the 8‐year‐old proband, who also had drug‐responsive absences associated with attention‐deficit/hyperactivity disorder. His 52‐year‐old father, who had focal epilepsy since childhood, developed paraparesis related to a reversible myelitis associated with hypoglycorrhachia. Molecular study detected a novel heterozygous missense variant (c.446C>T) in exon 4 of *SLC2A1* (NM: 006516.2) that cosegregated with the illness. This variant causes an amino acid replacement (p.Pro149Leu) at the fourth transmembrane segment of GLUT1, an important domain located at its catalytic core.

**Conclusions:**

Our study illustrates the extremely heterogenous phenotypes in familial GLUT1‐DS, ranging from milder classic phenotypes to more subtle neurological disorder including paraparesis. This novel *SLC2A1* variant (c.446C>T) provides new insight into the pathophysiology of GLUT1‐DS.

## INTRODUCTION

Glucose transporter 1 (GLUT1) deficiency syndrome (GLUT1‐DS) results from impaired glucose transport into the brain, due to mutations of the *SLC2A1* gene on chromosome 1, encoding GLUT1. The GLUT1‐DS “classical phenotype” includes developmental delay, intellectual disability (ID), movement disorders, ataxia, epilepsy, and microcephaly [1, 2]. Since its first description, a broader spectrum of GLUT1‐DS phenotypes has increasingly been recognized, and hypoglycorrhachia is a key diagnostic feature [3, 4, 5]. In GLUT1‐DS patients, especially those with epilepsy or movement disorders, ketogenic diet may achieve clinical benefit [2].

GLUT1‐DS usually presents as sporadic disease, with de novo *SLC2A1* variants leading to haploinsufficiency and conferring symptomatic heterozygosity. Approximately 10% of them are inherited in an autosomal dominant pattern, whereas an autosomal recessive pattern was described rarely [6, 7].

Among *SLC2A1* pathogenic variants, approximately 300 nonsense, frameshift, and splice‐sites variants result in 50% loss of function of GLUT1 and determine classical phenotype [2, 3, 6]. Conversely, missense variants with 50%–70% GLUT1 residual function lead more often to mild‐to‐moderate phenotypes. However, the genotype–phenotype correlation remained elusive [2, 3, 6].

Here, we report a large five‐generation family segregating a novel *SLC2A1* missense variant and aim to better define mild heterogeneous and atypical presentations consistent with GLUT1‐DS.

## MATERIALS AND METHODS

**FIGURE 1:**
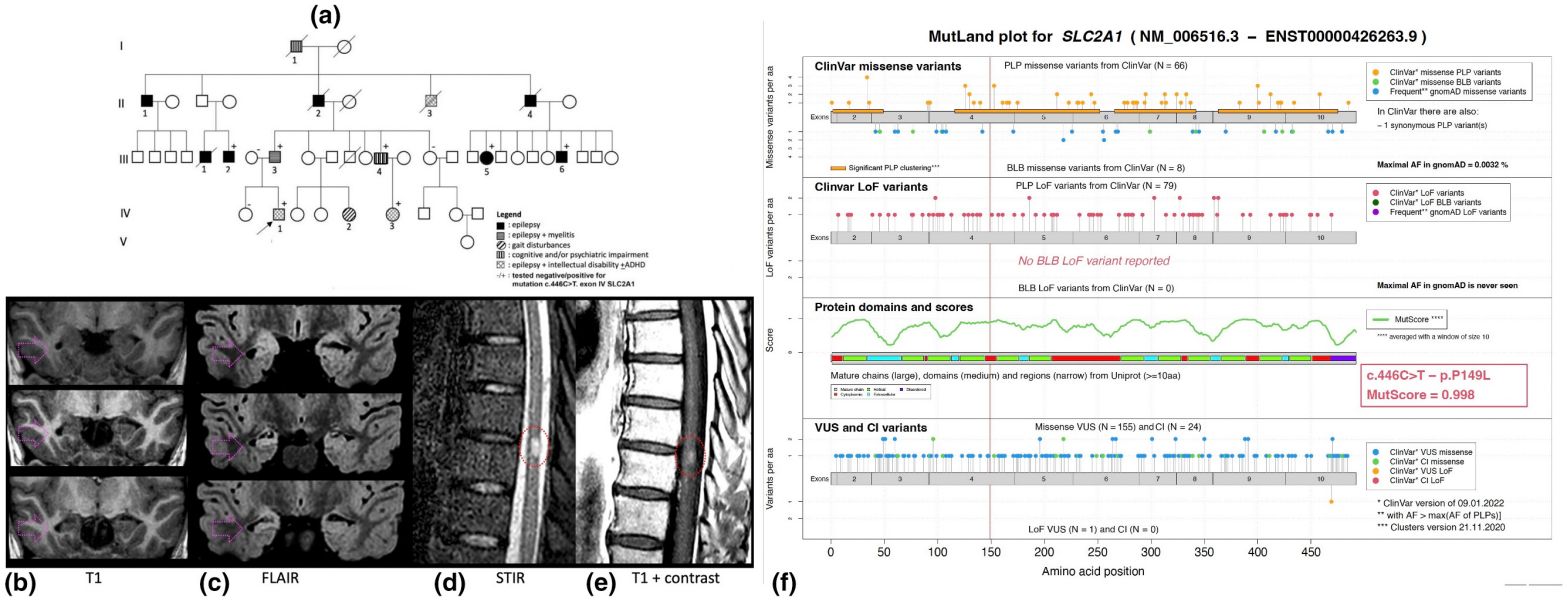
(a) Pedigree of the family. (b–e) In the proband, brain magnetic resonance imaging (MRI) gave evidence of atrophy (b) and hyperintensities (c) of the right hippocampal formation. In the father, spinal cord MRI demonstrated hyperintense lesion at T8/T9 level on short tau inversion recovery (STIR) sequences (d) with contrast enhancement on T1 sequences (e). (f) Visual presentation of MutScore prediction for *SLC2A1* (https://iob‐genetic.shinyapps.io/mutscore). All variants detected in ClinVar were reported: pathogenic and likely pathogenic (PLP), benign and likely benign (BLB), uncertain significance (VUS), and conflicting interpretation (CI). Our c.446C>T (p.P149L) variant is highlighted with red. aa, aminoacid; ADHD, attention‐deficit/hyperactivity disorder; AF, allelic frequency; FLAIR, fluid‐attenuated inversion recovery; LoF, loss of function.

The family (Figure 1a) originated in Calabria, southern Italy, and contained 14 (nine living) affected members (six men, mean age = 38.66 ± 28.05 years). The clinical data were compiled from detailed accounts given by family members at the time of investigation and from the patients' medical records (Table 1). Six of nine living affected members underwent a comprehensive evaluation, the procedures of which were reported elsewhere [8]. The diagnosis of epilepsy was based on the criteria of the International League Against Epilepsy [9].

**TABLE 1:** Clinical characterization of family members with glucose transporter 1 deficiency syndrome.

Individual	IV‐1	IV‐2	IV‐3	III‐1	III‐2	III‐3	III‐4	III‐5	III‐6	II‐1	II‐2	II‐3	II‐4
Gender	M	F	F	M	M	M	M	F	M	M	M	M	M
Age, years	8	16	12	Dead at 47	53	53	51	60	58	80	Dead at 78 years	Dead at 78 years	Dead at 78 years
Age at onset, years	4	5	6	Adolescence	Adolescence	12	50	Adulthood	Adolescence	Adulthood	Adulthood	Adulthood	Adulthood
Disease duration, years	3	11	6	N/A	N/A	41	1	N/A	N/A	N/A	N/A	N/A	N/A
Cognitive assessment	MID	Normal	MID	N/A	Normal	Normal	MID	MID	No	Normal	Normal	MID	Normal
Psychiatric symptoms	ADHD	No	No	Mood changes, psychosis	No	No	No	Mood changes, psychosis	Mood changes, psychosis	No	No	No	No
Gait disturbances	No	Yes	No	No	No	Yes	No	No	No	No	No	No	No
Epilepsy	CAE	No	CAE	FE	FE	FE	No	FE	No	FE	FE	FE	FE
EEG	3‐Hz GSW	Normal	3‐Hz GSW	N/A	N/A	Unremarkable	Normal	N/A	Unremarkable	N/A	N/A	N/A	N/A
Brain MRI	Right HS	N/A	Unremarkable	Unremarkable	Unremarkable	Unremarkable	Unremarkable	Unremarkable	Unremarkable	N/A	N/A	N/A	N/A
Glucose CFS‐to‐blood ratio	N/A	N/A	N/A	N/A	N/A	0.40	N/A	N/A	N/A	N/A	N/A	N/A	N/A
Genetic testing	+	+	+	N/A	+	+	+	+	+	N/A	N/A	N/A	N/A
ASM	Ethosuximide	No	Valproate	N/A	Levetiracetam	Carbamazepine	No	Phenobarbital	Oxcarbazepine	Phenobarbital	Levetiracetam	Levetiracetam	Carbamazepine

Abbreviations: ADHD, attention‐deficit/hyperactivity disorder; ASM, antiseizure medication; CAE, childhood absence epilepsy; CSF, cerebrospinal fluid; EEG, electroencephalography; F, female; FE, focal epilepsy; GSW, generalized spike‐waves; HS, hippocampal sclerosis; M, male; MID, mild intellectual disability; N/A, not available.

### Genetic analysis

Genetic analysis is summarized in Appendix S1. Molecular karyotyping (array comparative genomic hybridization [array‐CGH]) was conducted on peripheral blood DNA from the proband. Afterward, an exome sequencing panel (ES) of 72 genes for epilepsy was applied for the secondary bioinformatic analysis on the Sophia DDM online platform for automatic annotation and variant filtering.

We interpreted the identified variants according to several in silico tools (Sophia, VarSome), population databases (gnomAD v4.0), and disease database (ClinVar). Thus, we applied American College of Medical Genetics and Genomics (ACMG) criteria to classify the variant [10]. Sanger sequencing confirmed the variant in the proband, parents, and other family members.

### Standard protocol approvals, registrations, and patient consents

This study was approved by our institution's ethics committee and performed in accordance with the ethical standards laid down in the 1964 Declaration of Helsinki and its later amendments. We obtained patients' informed consent. Data were treated according to the European regulation General Data Protection Regulation n. 2016/679.

## RESULTS

Pregnancy, delivery, and the neonatal period were uneventful in all living members. The 8‐year‐old proband (IV‐1) developed, at age 4 years, childhood absence epilepsy (CAE) with attention‐deficit/hyperactivity disorder (ADHD) and mild ID (Wechsler Intelligence Scale for Children edition IV total intelligence quotient [IQ] = 85–95). Electroencephalography (EEG) revealed 3‐Hz generalized spike–wave discharges on normal background activity. Brain magnetic resonance imaging (MRI) detected right hippocampal sclerosis (HS; Figure 1b,c). In the past 18 months, ethosuximide (1 g/day) had been effective. The 51‐year‐old father (Patient III‐3) developed focal occipital epilepsy at age 12 years, well treated with carbamazepine (600 mg/day). At the age of 46 years, he developed acute self‐limiting paraparesis. He had no other comorbidities or prodromal illness. Spine MRI revealed increased T2 signal in the spinal cord with contrast enhancement at the T8–T9 level (Figure 1d,e). An extensive laboratory and cerebrospinal fluid (CSF) workup ruled out infectious, toxic, metabolic, and immune‐mediated disorders. Oligoclonal bands, anti‐aquaporin‐4‐IgG, and anti‐myelin‐oligodendrocyte‐glycoprotein‐IgG were absent on serum and CSF. Brain MRI, electromyography, and nerve conduction studies were not contributory. At 38‐month follow‐up, neurological examination showed a mild gait disturbance; EEG, and brain and spine MRI were all unremarkable. We retrospectively calculated the glucose CSF‐to‐blood ratio and found a value of 0.40 (fasting glucose = 106 mg/dL, normal range = 74–106 mg/dL; CSF glucose = 45 mg/dL, normal range = 40–75), with normal lactate.

Individual I‐1 died at the age 83 years; he had mild ID and developed cognitive decline later in life. Individual II‐1 is still alive at the age 80 years, whereas individuals II‐2, II‐3, and II‐4 died at the ages of 78, 77, and 82 years respectively. All had an almost identical epilepsy phenotype consisting of focal (head and eye version) to bilateral tonic–clonic attacks, starting in adulthood. Only individual II‐3 had mild ID. The remaining three had normal intelligence (based on the clinical impression of the relatives). Individuals III‐1, who died at 47 years, III‐2, III‐5, and III‐6, aged 53, 60, and 62 years respectively, had focal seizures with head and eye version, viscerosensory auras, and occasional evolution into bilateral tonic–clonic attacks, starting in adulthood, with mild‐to‐moderate psychiatric disturbances in all but individual III‐2.

Individual III‐4, aged 50 years, had mild short‐term and working‐memory deficits on cognitive assessment with no impact on daily life activities and unremarkable remaining neurological examination. He also had normal EEG and brain MRI. His 12‐year‐old daughter (IV‐3) presented CAE at age 8 years, with mild ID (IQ = 90) and unremarkable neurological examination and brain MRI. EEG showed generalized sharp waves at 3 Hz on normal background activity. Ethosuximide (500 mg/day) was effective. Individual IV‐2, 16 years of age, developed gait disturbances since age 5 years with no ID or psychiatric disturbances. In patients IV‐1 and IV‐3, the ketogenic diet improved behavior and seizures, but it was stopped after 4 months because of noncompliance.

### Genetic analysis

In the proband, array‐CGH was unremarkable. The ES panel revealed a heterozygous missense variant c.446C>T (p. Pro149Leu) in exon 4 of *SLC2A1* (NM_006516.2), inherited from his father (III‐3) and confirmed by Sanger sequencing. Exon 4 is a mutational hotspot, harboring approximately one third of *SLC2A1* mutations in GLUT1‐DS [4]. It encodes for a highly conserved domain, among all known sugar transporters from different species, TM4, at the catalytic core. The subsequent aminoacidic substitution from a proline to a leucine alters GLUT1 structure and function. This c.446C>T variant has not been previously reported in ClinVar, in Genome Aggregation Database version 4.0, or in population studies (ESP5400: NHLBI Exome Sequencing Project and 1000 Genomes Project).

Thus, according to the ACMG, its location in a mutational hotspot with critical and well‐established functional domain (PM1), the absence from controls (PM2_Supporting), its cosegregation with disease in multiple affected family members (PP1_Moderate), and in silico tools (aggregated metaRNN score = 0.9918, pathogenic strong) that unanimously support the deleterious effects of the aminoacidic replacement on GLUT1 structure and function (PP3) demonstrated that this novel variant can be classified as a likely pathogenic variant (Figure 1f, Appendix S1) [10].

## DISCUSSION

This family extends the clinical spectrum of GLUT1‐DS, ranging from milder classic phenotypes to more subtle neurological disorder carriers, further strengthening the idea that familial GLUT1‐DS is less severe than the sporadic form [2, 3, 4, 5, 6, 7, 8, 9, 10, 11]. Different from the classical phenotype, our family depicted heterogeneous association of mild, drug‐responsive epilepsies and subtle cognitive, psychiatric, and gait disturbances. In the proband who developed typical absences and ADHD, MRI revealed HS. We are unaware of other reports of HS in GLUT1‐DS. Several factors may contribute to HS, such as prolonged febrile seizures, and repetitive generalized or focal seizures, especially during status epilepticus [12]. In our case, we have no definite explanation of this finding, as the proband did not experience febrile, prolonged, or repetitive seizures. Interestingly, other than brain–blood barrier, GLUT1 receptors are widely but heterogeneously expressed in neurons, astrocytes, and oligodendroglial and microglial cells [13]. Both preclinical and human studies demonstrated a susceptibility of mesial temporal cortex to GLUT1 defect. The transgenic mouse model of GLUT1‐DS showed decreased hippocampal volume, whereas ^18^F‐fluorodeoxyglucose positron emission tomography quantitative analysis demonstrated a more prominent hypometabolism over the mesial temporal cortex, suggesting its susceptibility to GLUT1 defect [13]. Thus, these findings suggest a direct causal relationship between HS and GLUT1 defect [13].

Overall, appreciation of atypical GLUT1‐DS phenotypes is important from a diagnostic perspective and might also offer insights into the mechanisms by which different variants cause disease. In this regard, the proband's father developed an unusual phenotype with focal epilepsy and self‐limiting acute paraparesis. Only few reports have addressed the causative role of *SLC2A1* mutations in spastic paraparesis, direct or more often combined with complex phenotype, including paroxysmal exercise‐induced dyskinesia, ID, and epilepsy [11, 14, 15]. To our knowledge, this is the first case of self‐limiting paraparesis in a subject carrying an *SLC2A1* missense variant. Thus, these findings emphasize the importance of considering GLUT1‐DS in the diagnostic workup of patients with transient acute neurological symptoms [11], especially if associated with prior epilepsy. Although we cannot rule out the incidental nature of this evidence, the glucose CSF‐to‐blood ratio below the cutoff and genetic analysis are convincing that the acute paraparesis is part of GLUT1‐DS [3].

The heterozygous missense variant (c.446C>T) is in a critical domain at the catalytic core of GLUT1 and has a deleterious effect on structure and function [5]. Missense variants constitute approximately 40% of *SLC2A1* mutations and are often related to mild‐to‐moderate phenotype [3, 10]. Probably, the extremely heterogeneous phenotypes of family members carrying the same mutation indicate that secondary genes and other modifying factors may modulate the expression level of GLUT1. It is also possible that the wild‐type *SLC2A1* allele might modify the phenotypic expression of the mutant allele and thus contribute to heterogeneity in affected GLUT1‐DS families.

## AUTHOR CONTRIBUTIONS

**Alessia Giugno:** Writing – original draft; conceptualization; methodology; formal analysis; data curation. **Elena Falcone:** Formal analysis; data curation; writing – original draft. **Francesco Fortunato:** Writing – original draft; data curation. **Ilaria Sammarra:** Data curation; writing – original draft. **Radha Procopio:** Data curation; formal analysis. **Monica Gagliardi:** Data curation; formal analysis. **Alessia Bauleo:** Formal analysis; data curation. **Laura de Stefano:** Data curation; formal analysis. **Iolanda Martino:** Formal analysis; data curation. **Antonio Gambardella:** Writing – review and editing; supervision.

## FUNDING INFORMATION

This work was funded by #NEXTGENERATION (NGEU) and by the Ministry of University and Research, National Recovery and Resilience Plan, project MNESYS (PE0000006; A Multiscale Integrated Approach to the Study of the Nervous System in Health and Disease; DN.1553 11.10.2022).

## CONFLICT OF INTEREST STATEMENT

The authors have stated explicitly that there are no conflicts of interest in connection with this article.

## ETHICAL STANDARDS

This survey has been approved by our institution's ethics committee and has been performed in accordance with the ethical standards laid down in the 1964 Declaration of Helsinki and its later amendments. We obtained patients' informed consent, and data were treated according to the European General Data Protection Regulation, n. 2016/679.

## Supporting information


Appendix S1.

## Data Availability

The data that support the findings of this study are available on request from the corresponding author. The data are not publicly available due to privacy concerns.
