# Supplementary material for: Glucose transporter‐1 deficiency syndrome with extreme phenotypic variability in a five‐generation family carrying a novel SLC2A1 variant
Source: Eur J Neurol. 2024 May 27;31(8):e16325. doi: 10.1111/ene.16325 (PMC11235872; doi:10.1111/ene.16325)
Supplement: Supplementary file 1 — Appendix S1. [file ENE-31-e16325-s001.docx]

**Glut1-deficiency syndrome with extreme phenotypic variability in a five-generation family carrying a novel SCL2A1 variant**

DNA from probands and relatives was extracted from peripheral blood using standard procedures by Qiagen DNA easy spin-column kits on a semi-automatic QIAcube instrument (Qiagen).

Libraries preparation was performed using Clinical Exome Solution (CES, Sophia Genetics SA, Saint-Sulpice, Switzerland) kit, covering 4490 genes with known disease-causing genes. Thereaftersample quality and quantity controls were assessed using DNA D1000 kit and a Tape Station 4100 respectively (Agilent Technologies, Santa Clara, CA, USA). Massively parallel sequencing was performed on the MiSeq Illumina platform with a read length of 300 x 2. Data processing, filtering and base calling was performed using Real-Time Analysis (RTA) software integrated in the MiSeq instrument (Illumina).

We restricted CES data to 72 genes implicated in epilepsy (virtual gene panel, Table 1), with raw reds aligned to the human reference genome (GRCh37/ hg19). Variant filtering and interpretations were performed on the Sophia DDM™ platform (Sophia Genetics SA) according to American College of Medical Genetics and Genomics criteria. Sanger sequencing was performed to confirm variant in the proband and its segregation in parents and other family members.

**Figure**

**(A)** NGS showed a heterozygous variant, (c.446C>T), (p.Pro149Leu) in exon 4 in of the *SCL2A1* (NM_006516.2) located within the TM4 segment binding domain of GLUT1 transporter.

**(B)** Sanger sequencing confirmed this variant (c.446C>T), leading to an amino acids substitution (p.Pro149Leu) TM4.

**(C)** Visual presentation of MutScore (https://iob-genetic.shinyapps.io/mutscore) prediction for SLC2A1. All variants detected in ClinVar were reported: pathogenic and likely pathogenic (PLP), benign and likely benign (BLB), uncertain significance (VUS) and conflicting interpretation (CI). Our c.446C>T (p.P149L) variant is highlighted with red line.

**(A)**

**(B)**

**Mother**

**Proband**


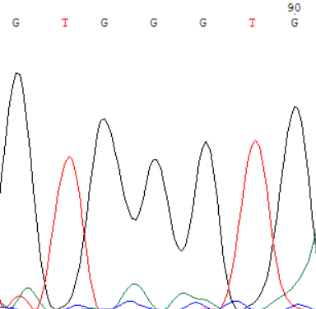

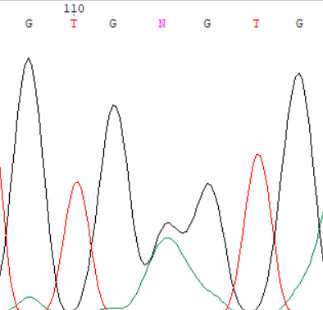


**Sister**

**Father**


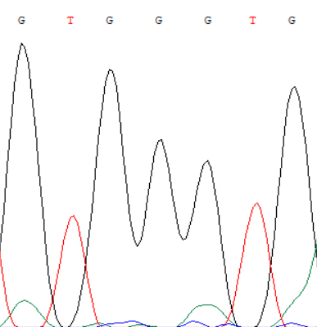

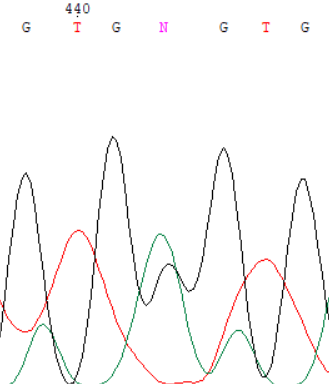


**(C)**


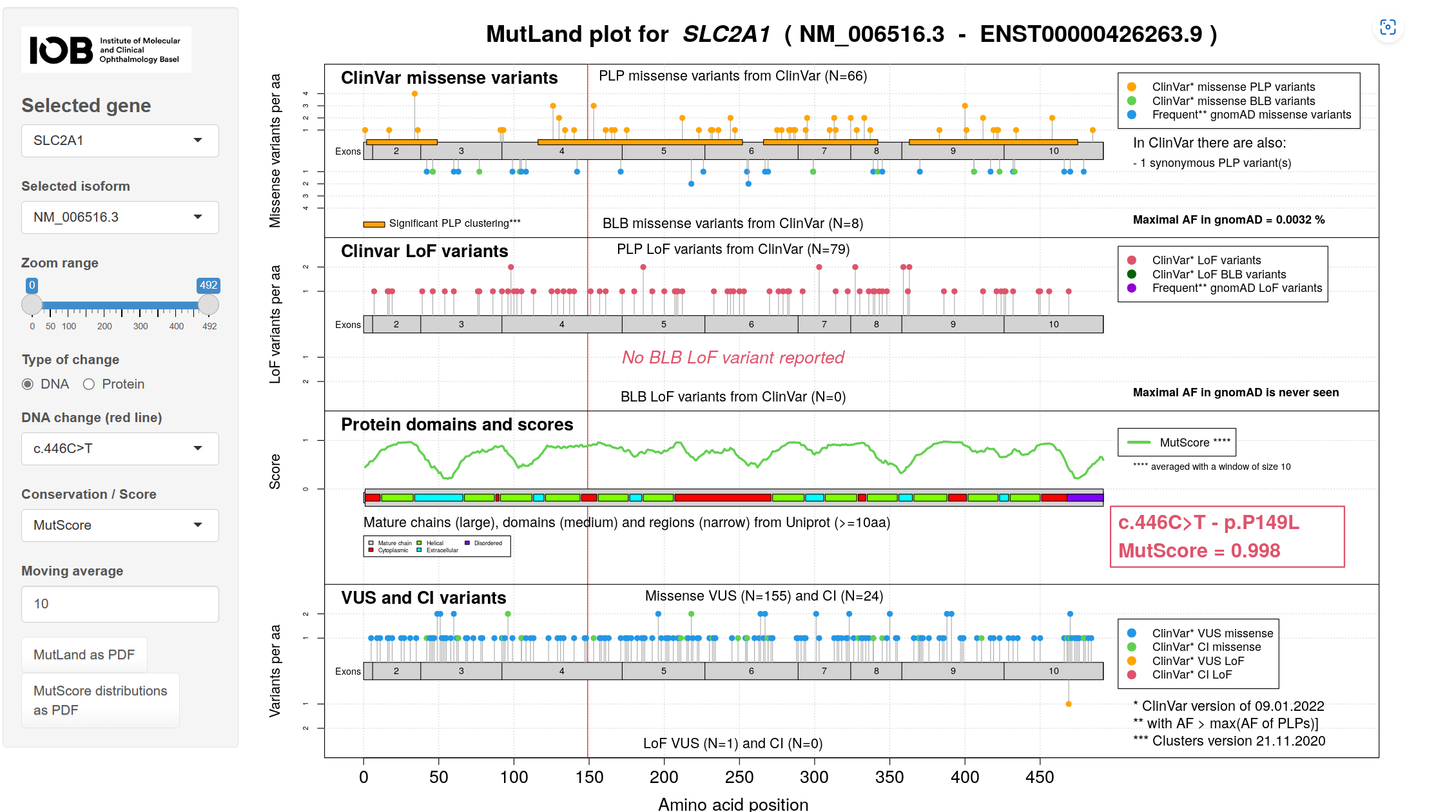


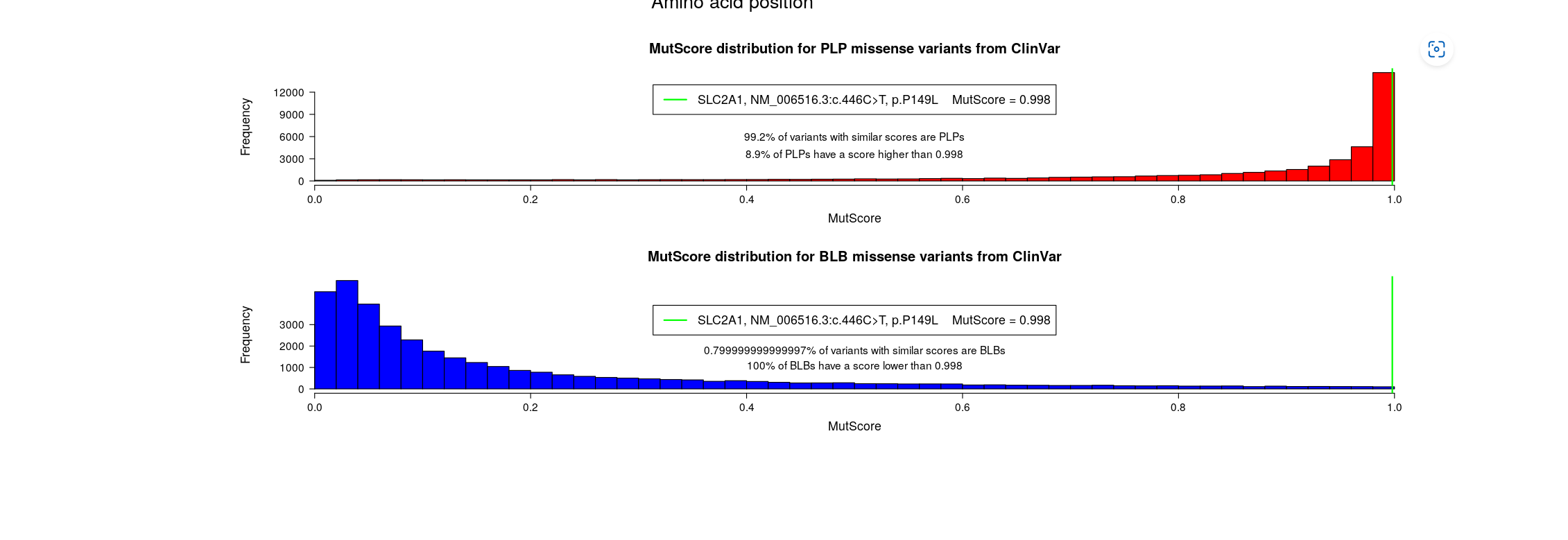


**Table 1. Epilepsy gene panel (72 genes)**

*AARS, ADSL, ALDH7A1, ARX, ASAH1, ATP1A2, ATP6AP2, CACNB4, CDKL5, CHRNA2, CHRNA4, CHRNB2, CLN3, CLN5, CLN6, CLN8, CNTNAP2, CSTB, CTSD, DNAJC5, EFHC1, EPM2A, FOLR1, FOXG1, GABRA1, GABRG2, GAMT, GATM, GOSR2, GRIN2A, GRIN2B, KCNJ10, KCNQ2, KCNQ3, KCTD7, LGI1, LIAS, MAGI2, MBD5, MECP2, MEF2C, MFSD8, NHLRC1, NRXN1, PCDH19, PNKP, PNPO, POLG, PPT1, PRICKLE1, PRICKLE2, PRRT2, SCARB2, SCN1A, SCN1B, SCN2A, SCN8A, SLC25A22, SLC2A1, SLC9A6, SPTAN1, SRPX2, STXBP1, SYN1, TBC1D24, TCF4, TPP1, TSC1, TSC2, UBE3A, WWOX, ZEB2*
